# Potential Climate Change Effects on the Habitat of Antarctic Krill in the Weddell Quadrant of the Southern Ocean

**DOI:** 10.1371/journal.pone.0072246

**Published:** 2013-08-21

**Authors:** Simeon L. Hill, Tony Phillips, Angus Atkinson

**Affiliations:** 1 British Antarctic Survey, Natural Environment Research Council, Cambridge, United Kingdom; 2 Plymouth Marine Laboratory, Plymouth, United Kingdom; Institute of Marine Research, Norway

## Abstract

Antarctic krill is a cold water species, an increasingly important fishery resource and a major prey item for many fish, birds and mammals in the Southern Ocean. The fishery and the summer foraging sites of many of these predators are concentrated between 0° and 90°W. Parts of this quadrant have experienced recent localised sea surface warming of up to 0.2°C per decade, and projections suggest that further widespread warming of 0.27° to 1.08°C will occur by the late 21^st^ century. We assessed the potential influence of this projected warming on Antarctic krill habitat with a statistical model that links growth to temperature and chlorophyll concentration. The results divide the quadrant into two zones: a band around the Antarctic Circumpolar Current in which habitat quality is particularly vulnerable to warming, and a southern area which is relatively insensitive. Our analysis suggests that the direct effects of warming could reduce the area of growth habitat by up to 20%. The reduction in growth habitat within the range of predators, such as Antarctic fur seals, that forage from breeding sites on South Georgia could be up to 55%, and the habitat’s ability to support Antarctic krill biomass production within this range could be reduced by up to 68%. Sensitivity analysis suggests that the effects of a 50% change in summer chlorophyll concentration could be more significant than the direct effects of warming. A reduction in primary production could lead to further habitat degradation but, even if chlorophyll increased by 50%, projected warming would still cause some degradation of the habitat accessible to predators. While there is considerable uncertainty in these projections, they suggest that future climate change could have a significant negative effect on Antarctic krill growth habitat and, consequently, on Southern Ocean biodiversity and ecosystem services.

## Introduction

Climate warming is already producing complex spatial and seasonal changes in the Earth’s habitats and ecosystems [1], [2]. Warming is expected to increase significantly over the 21^st^ Century [3], leading to ecosystem change and potentially severe socioeconomic consequences [4]. Observed changes in the Southern Ocean include localised sea ice loss [5] and increases in summer sea surface temperatures (SSTs) of 1°C over 5 decades near the western Antarctic Peninsula [6] and 0.9°C over 8 decades at South Georgia [7]. Previous studies have identified potential relationships between climate-related variables (sea temperature, ice cover and pH) and the recruitment, survival, growth and distribution of the crustacean Antarctic krill, *Euphausia superba* [8], [9], [10]. Antarctic krill is a characteristic species of the Southern Ocean and exists within a narrow band of cold temperatures (up to ∼5°C) [11], [12], [13]. It is an increasingly important fishery resource and a major prey item for a diverse suite of predators including whales, penguins, seals and fish [14], [15], [16], [17], [18], [19]. The role of Antarctic krill in supporting predators might be more significant than that of any comparable species elsewhere in the world’s oceans [16].

**Figure 1:**
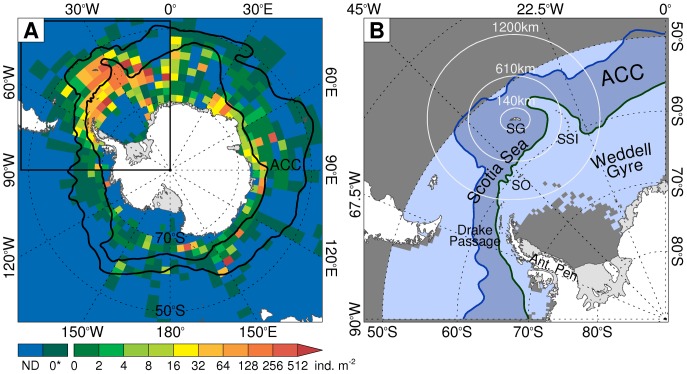
The distribution of Antarctic krill and the study area. (A) The observed distribution of Antarctic krill (individuals.m^−2^ within each 5° longitude by 2° latitude grid cell, ND = no data, 0* = no Antarctic krill recorded in the available data) from [12]. (Inset & B) The study area, showing the Antarctic Circumpolar Current (ACC), which is bounded to the north by the Antarctic Polar Front and to the south by the Southern boundary of the ACC (Positions from [36]). The concentric distances from South Georgia (SG) indicate the approximate foraging ranges of representative predators of Antarctic krill: Antarctic fur seals (140 km), Wandering albatrosses (610 km) and Grey-headed albatrosses (1200 km). Areas north of 50°S (shaded grey) were not included in the study. Ant. Pen = Antarctic Peninsula, SO = South Orkney Islands, SSI = South Sandwich Islands.

Antarctic krill has an estimated biomass in excess of 2×10^8^t [18], [20], about one-quarter of which is concentrated in about 10% of its total habitat area, specifically the Scotia Sea and southern Drake Passage (Fig. 1) [11], [12], [21]. This is where many air breathing vertebrates congregate to feed on Antarctic krill and rear offspring on islands such as South Georgia [18], [22]. For example, about 95%, 50% and 25% [23], [24], [25] of the global populations of Antarctic fur seals (*Arctocephalus gazella*), grey headed albatrosses (*Thalassarche chrysostoma*) and wandering albatrosses (*Diomedea exulans*) breed at South Georgia where Antarctic krill constitute approximately 85%, 76% and 12% of their respective diets [15], [26]. Antarctic krill is also an abundant fishery resource which, according to the Food and Agriculture Organisation of the United Nations, is underexploited [27]. The potential harvest from the Scotia Sea and southern Drake Passage is equivalent to 7% of current global marine fisheries production [28].

It is important to evaluate how further environmental change might affect Antarctic krill and consequently the biodiversity and ecosystem services of the Southern Ocean. In this study, we examine some of these effects by assessing potential changes in the habitat’s ability to support Antarctic krill growth. Growth represents the accumulation of resources within an individual or population and is therefore a valuable indicator of overall habitat quality [29]. Individual Antarctic krill can increase their body mass by up to a factor of four within a single growing season [20] and this production of new biomass supports predators and the fishery. We assess potential habitat changes over the 21^st^ century by combining a statistical model of Antarctic krill growth [30] with projected SST changes from the Coupled Model Intercomparison Project Phase 5 (CMIP5) multi-model ensemble [31]. The statistical model links growth to body size, SST and food availability. We focus on the quadrant between 0° and 90°W (also known as the Weddell Quadrant [32]), which encompasses the Scotia Sea and southern Drake Passage, and we assess potential changes within the ranges of predators foraging from South Georgia in more detail.

## Methods

### Models and Metrics

An assessment of various models for evaluating Antarctic krill growth habitat on the basis of temperature [33] favoured the use of statistical models [30], [34]. These models are based on the observed growth rates of Antarctic krill caught in a wide range of environmental conditions. We used one such model [30], [12] that relates the daily increase in Antarctic krill length (daily growth rate: *DGR*, mm.d^−1^) to sea surface temperature (*SST*, °C), food availability indicated by chlorophyll-a concentration (*CHL*, mg.m^−3^), and starting length (*L*, mm):
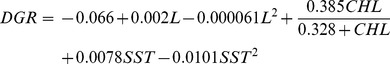
(1)


The following relationship, which was derived from the same data as equation 1 [30], converts individual Antarctic krill length (mm) to dry mass, *M* (mg):
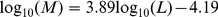
(2)


Previous studies [12], [20] have used equations 1 and 2 to estimate Gross Growth Potential (GGP) based on spatially-resolved, monthly averages of *SST* and *CHL*. GGP is the model-predicted dry mass of an individual Antarctic krill at the end of the summer growth season divided by its dry mass at the beginning of the season. GGP is therefore a unitless quantity that indicates the habitat’s ability to support Antarctic krill growth.

The CMIP5 dataset [31] provides the results of climate simulations from multiple climate models. The variations in important factors such as greenhouse gases and aerosols which were used to drive simulations to 2005 were observed values, whereas simulations from 2006 were forced with Representative Concentration Pathways (RCPs). These RCPs include representations of potential changes to factors such as greenhouse gas and air pollutant emissions and land-use, and are named according to projected radiative forcing in the year 2100 [35]. RCP2.6 has peak radiative forcing of ∼3 W.m^−2^ in the first half of the 21^st^ century, falling to ∼2.6 W.m^−2^ by 2100. In this scenario, aggressive mitigation results in negative net greenhouse gas emissions by the end of the century. Under RCP4.5, greenhouse gas emissions rise until around 2040 before falling below those for the year 2000 by the end of the century, and radiative forcing stabilizes at ∼4.5 W.m^−2^ around 2100. Under RCP8.5, radiative forcing reaches 8.5 W.m^−2^ in 2100 and continues to rise [31], [35].

We used results from RCP2.6, RCP4.5 and RCP8.5 to calculate projected 21^st^ Century SST changes for the Southern Ocean in the longitudinal quadrant 0°W to 90°W. We define the Southern Ocean as the marine area south of the Antarctic Polar Front (position defined in [36], 2008 update available from: data.aad.gov.au/aadc/metadata/metadata_redirect.cfm?md = /AMD/AU/southern_ocean_fronts). We explored the implications of projected SST change for spatially-resolved GGP in the same quadrant for the area south of 50°S (the study area). 50°S is the northern limit of Antarctic krill distribution [11], [12].

We used equation 1 to calculate the length increase per week for summer growing seasons in the current (2002–2011) and projection (2070–2099) periods. These calculations used monthly averages of *SST* and *CHL*. For weeks that straddled two months, we used weighted averages of *SST* and *CHL* from those two months. We updated the Antarctic krill starting length at the beginning of each week and converted the initial and final lengths to dry mass using equation 2. We then divided final mass by starting mass to estimate GGP.

To estimate current GGP, we used monthly estimates of current SST based on observations [37], which we label 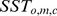. Subscript *o* indicates that the data are observations, *m* indicates the month and *c* indicates that the estimate is a climatology (i.e. a long term average). Climatologies are appropriate for variables, such as SST, that have high interannual variability [38]. Our estimates of projected GGP were based on estimates of projected SST, 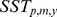, from the CMIP5 results, where the subscript *p* indicates that the data are projections and *y* indicates year. To correct for bias in model estimates of SST [38], we first converted projected SST into differences from an SST climatology representing current conditions in the same model, 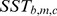, where subscript *b* indicates model baseline conditions. We then added the current climatology based on observations, 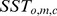, to calculate a bias-corrected estimate of projected SST, 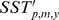:
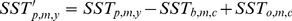
(3)


We used this bias-corrected SST estimate to calculate projected GGP.

The World Meteorological Organisation recommends calculating climatological conditions over 30 years (www.wmo.int/pages/prog/wcp/ccl/faqs.html). We therefore selected the period 1991–2020 for 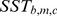 and we obtained 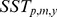 for each year 2070–2099. The climatological period for 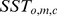 was restricted by data availability to 2002–2011. We obtained 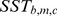 and 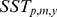 for each available model in the CMIP5 results. Within each RCP, SST estimates for each model were calculated as the mean of the estimates for all realisations available for that model. Consequently contributions from all models were given equal weight in across-model means, regardless of the number of realisations per model.

In addition to SST data, GGP estimation requires a starting length and *CHL* estimates. We used a starting length of 40 mm, which is the observed mean length for the postlarval population of Antarctic krill [20]. Following [20] we also considered starting lengths of 30 mm and 50 mm.

We used remote-sensed, spatially-resolved *CHL* estimates (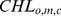) [39]. The climatological period for 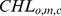 was restricted by data availability to 1997–2010. We varied these *CHL* estimates to assess the sensitivity of our results to assumptions about chlorophyll-a concentration. One study estimates that the Southern Ocean experienced a 10% decline in chlorophyll-a concentration over about 17 years between the 1980s and 1990s [40]. Linear extrapolation of this change suggests a 50% reduction over our eight decade projection period. We therefore decreased the *CHL* estimates by 50%. We also considered increases by the same amount.

We calculated spatially-resolved estimates of current GGP using 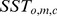 and 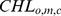, and spatially-resolved estimates of projected GGP for each year 2070–2099 for each available CMIP5 model in each of the three RCPs. Our GGP estimates for the period 2070–2099 were resolved to grid cell (1° longitude by 0.5° latitude), model and projection year. We averaged across years and models to derive a single estimate of projected GGP for each combination of grid cell, chlorophyll-a concentration, and RCP. We then subtracted the estimated current GGP (i.e. that calculated using observed SSTs, 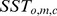, and observed chlorophyll-a concentrations, 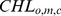) from projected GGP to estimate the GGP change between the current period and 2070–2099.

From our spatially-resolved GGP estimates, we calculated three spatially-aggregated metrics for each combination of chlorophyll-a concentration and RCP: (1) average GGP by year, (2) total GGP, and (3) growth area. Average GGP by year is the mean of those across-model, grid-cell-and-year-specific GGP estimates that were ≥1. GGP<1 indicates that the habitat does not support growth or maintenance of body size. It implies shrinkage resulting from starvation, which has been observed in Antarctic krill [41]. To estimate total GGP we first calculated, for each grid cell in each model, the across-year mean GGP for the period 2070–2099. We then calculated, for each model, the area-weighted sum of those resulting grid cell-specific estimates of GGP that were ≥1. Total GGP is the across-model mean of this sum. Weighting by grid cell area was necessary because this area changes with latitude. To estimate growth area we calculated, for each model, the total area of all grid cells in which the across-year mean GGP was ≥1. Growth area was the across-model mean of this sum.

We estimated projected change relative to current conditions in the form of relative GGP and relative growth area. We calculated these relative values by dividing total GGP and growth area by the equivalent metric calculated using observed SSTs, 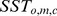, and chlorophyll-a concentrations, 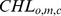. Our estimate of relative GGP excludes GGP values <1 and therefore does not include further degradation of habitat that did not initially support growth. It does, however, include such habitat becoming viable for growth. We calculated relative GGP and relative growth area for our entire study area, and within the foraging ranges of representative Antarctic krill predators foraging from South Georgia.

### Data

We obtained spatially-resolved monthly mean SST data at a nominal horizontal resolution of 9 km for the austral summer periods (December to March) from December 2002 to March 2011 from the archive of Aqua MODIS level 3 data [37], and chlorophyll-a concentration data for the austral summer periods from December 1997 to March 2010 from the archive of SeaWIFS data [39]. Both of these datasets are available through the NASA OceanColor website (*oceancolor.gsfc.nasa.gov*). We obtained spatially-resolved, monthly mean SST data for the period 1990–2100, for each selected RCP, from the output of multiple climate models which are available as part of the CMIP5 multi-model ensemble results [31]. The CMIP5 model data were downloaded from the distributed CMIP5 archive accessed via the Program for Climate Model Diagnosis and Intercomparison CMIP5 data portal (cmip-pcmdi.llnl.gov/cmip5/data_portal.html). We used the results that were available on 31 Jan 2012, including 14 sets of model results available for RCP2.6, 15 for RCP4.5 and 16 for RCP8.5 (Table 1). SST in these results is the average temperature of the surface layer of the modelled ocean. The depth of this layer is <20 m in all 16 models and exactly 10 m in 10 of them.

**Table 1:** The climate models used in this study.

Model name	Modelling group	Number of realisations			Global number of SST grid points		SST on regular grid?
		RCP2.6	RCP4.5	RCP8.5	Longitudinal	Latitudinal	
BCC-CSM1.1	Beijing Climate Center, China Meteorological Administration	1	1	1	360	232	No
CanESM2	Canadian Centre for Climate Modelling and Analysis	5	5	5	256	192	Yes
CNRM-CM5	Centre National de Recherches Météorologiques/Centre Européen deRecherche et Formation Avancée en Calcul Scientifique	1	1	5	362	292	No
CSIRO-Mk3.6.0	Commonwealth Scientific and Industrial Research Organization incollaboration with Queensland Climate Change Centre of Excellence	10	10	10	192	189	Yes
EC-EARTH	EC-EARTH consortium	2	9	9	362	292	No
GFDL-ESM2G	NOAA Geophysical Fluid Dynamics Laboratory	1		1	360	210	No
GISS-E2-R	NASA Goddard Institute for Space Studies	1	5	1	144	90	Yes
HadGEM2-CC	Met Office Hadley Centre (additional HadGEM2-ES realizationscontributed by Instituto Nacional de Pesquisas Espaciais)		1	3	360	216	Yes
HadGEM2-ES		4	3	4	360	216	Yes
INM-CM4	Institute for Numerical Mathematics		1	1	360	340	No
IPSL-CM5A-LR	Institut Pierre-Simon Laplace	3	4	4	182	149	No
IPSL-CM5A-MR		1	1	1	182	149	No
MIROC5	Atmosphere and Ocean Research Institute (The University of Tokyo),National Institute for Environmental Studies, and Japan Agencyfor Marine-Earth Science and Technology	3	3	3	256	224	No
MPI-ESM-LR	Max-Planck-Institut für Meteorologie (Max Planck Institute for Meteorology)	3	3	3	256	220	No
MRI-CGCM3	Meteorological Research Institute	1	1	1	360	368	No
NorESM1-M	Norwegian Climate Centre	1	1	1	320	384	No

The table lists the models used in this study, identifies the number of realisations (individual model runs) available for each of the three Representative Control Pathways (RCPs 2.6, 4.5 and 8.5), and specifies the spatial resolution and grid type for each model.

In addition to analysing habitat quality across the study area, we also extracted statistics for the areas within each of three concentric distances from South Georgia that indicate the summer foraging ranges of representative near, medium and long-range foragers. These distances were 140 km, 610 km, and 1200 km which respectively indicate the foraging ranges of Antarctic fur seals [42], wandering albatrosses [26] and grey headed albatrosses [26] during the summer offspring rearing period.

The data were initially available on different types of grid which we converted to a common grid of 1° longitude by 0.5° latitude. The remote-sensed SST and chlorophyll-a concentration data were both available on regular, fine-scale grids with a resolution of 5 arc minutes, so for each grid cell in the common grid we simply calculated the mean of the 72 constituent fine-scale grid cells. Some of the CMIP5 SST data were provided on a regular grid. We converted these data to the resolution of the common grid using bilinear interpolation [43]. For data not provided on a regular grid, we used all grid points to generate a Delaunay triangulation on an equirectangular projection [44], and we converted the data from this triangulation onto the common grid using linear interpolation [45]. We flagged as missing data any value on the common grid that was affected by a land point. The representation of coastline varies between models, so GGP estimates in coastal cells are informed by varying numbers of models. This does not affect any of our main conclusions.

The availability of remote-sensed SST and chlorophyll-a concentration data varies temporally due to the presence of cloud and ice cover, and cells with insufficient coverage appear as missing data in the Aqua MODIS and SeaWIFS monthly mean data products. Our objective was to achieve extensive spatial coverage with sufficient observations in each cell to provide representative monthly SST and chlorophyll-a concentration estimates for the summer growth season. We achieved a suitable balance of spatial and temporal coverage by including only those 1° by 0.5° cells for which data were available for a minimum of 20% of the initial fine-scale grid cells per month for at least 3 years during the climatology period. To maximise spatial data coverage, we constructed the climatologies from the full period of data availability for each data type separately, and restricted the analysis to the period January to March. The majority of krill growth occurs between December and March [11], and previous studies of current habitat quality have used this four month period but consequently had less spatial coverage [12], [20]. The application of these criteria defined the areas for inclusion and exclusion of data in our calculations, which we applied consistently to each data set that we used.

## Results

The results presented in this section are for an assumed Antarctic krill starting length of 40 mm. The Supporting Information (Figs. S1, S2 and S3) compares results for different starting lengths (30 mm, 40 mm and 50 mm).

**Figure 2:**
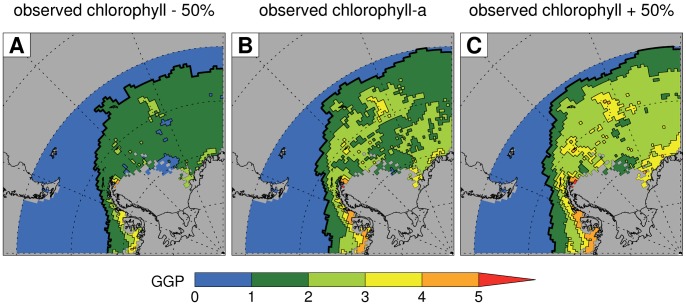
Current Antarctic krill summer growth habitat quality and sensitivity to chlorophyll concentration. (A) Gross Growth Potential (GGP, a unitless quantity which indicates the potential proportional increase in the mass of an individual Antarctic krill during a single summer and is therefore a measure of habitat quality) calculated for an individual with a starting length of 40 mm using observed SSTs (for the period 2002–2011), and observed chlorophyll-a concentrations (for the period 1997–2010) reduced by 50%. (B) Estimated current GGP calculated using observed SSTs, and observed chlorophyll-a concentrations. (C) Estimated current GGP calculated using observed SSTs, and observed chlorophyll-a concentrations increased by 50%. The spatial resolution is 1° longitude by 0.5° latitude and the thick black line indicates the northern extent of the growth area (the habitat that supports Antarctic krill growth with the relevant chlorophyll-a concentration). Thus, the thick black line in (B) delimits the current growth area.

The growth model correctly identified the warmer waters north of the Antarctic Polar Front as unable to support Antarctic krill growth (Fig. 2). There was considerable spatial structure in current GGP estimates for the study area, including patches of elevated habitat quality along the coast of the Antarctic continent and around the South Orkney and South Sandwich islands. These patterns were less distinct but still apparent with changed chlorophyll-a concentrations. Fig. 2 shows extensive areas in the southern Weddell Sea and along the coast of the Antarctic Peninsula for which we did not calculate GGP because of low data availability due to frequent ice cover.

**Figure 3:**
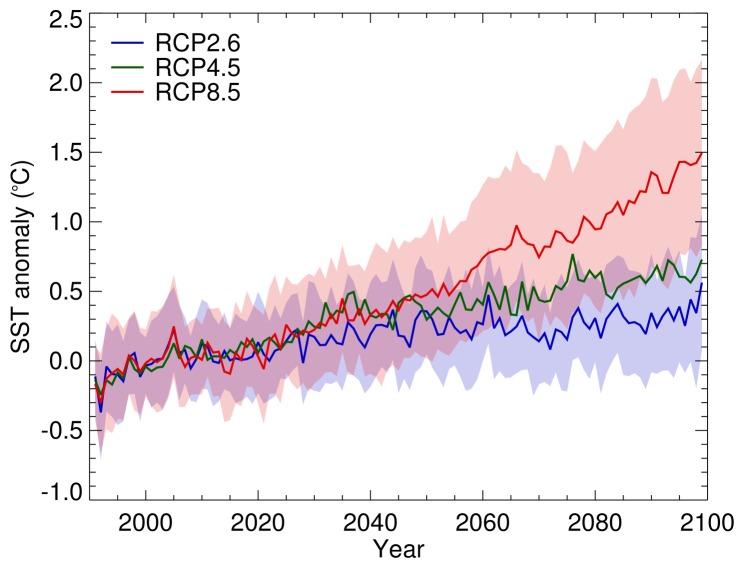
Projected 21^st^ Century summer surface warming of the Southern Ocean between 0° and 90°W. Projected summer (January to March) sea surface temperature (SST) anomaly for the region between 0° and 90°W and south of the Antarctic Polar Front (Fig. 1). The SST anomaly is the within-year mean of spatially-resolved summer SSTs for a specific model realisation minus the 1991–2020 mean of spatially-resolved summer SSTs for the same model realisation. The coloured lines indicate the mean SST anomaly for 1991–2099 across all available models (Table 1) for each of three Representative Control Pathways (RCPs 2.6, 4.5 and 8.5) and the shaded envelopes indicate the between-realisation standard deviation for RCPs 2.6 and 8.5.

Monthly climatological SSTs from CMIP5 models for the period 1991–2020 were, on average, 2.04°C warmer than SST estimates for 2002–2011 from Aqua MODIS data, but there was reasonable spatial correlation between the two datasets (r = 0.954). The mean projected summer SST warming for the area south of the Antarctic Polar Front between 1991–2020 and 2070–2099 was 0.27°C, 0.56°C and 1.08°C for RCP2.6, RCP4.5 and RCP8.5 respectively. These estimates varied between years and between models (Fig. 3).

**Figure 4:**
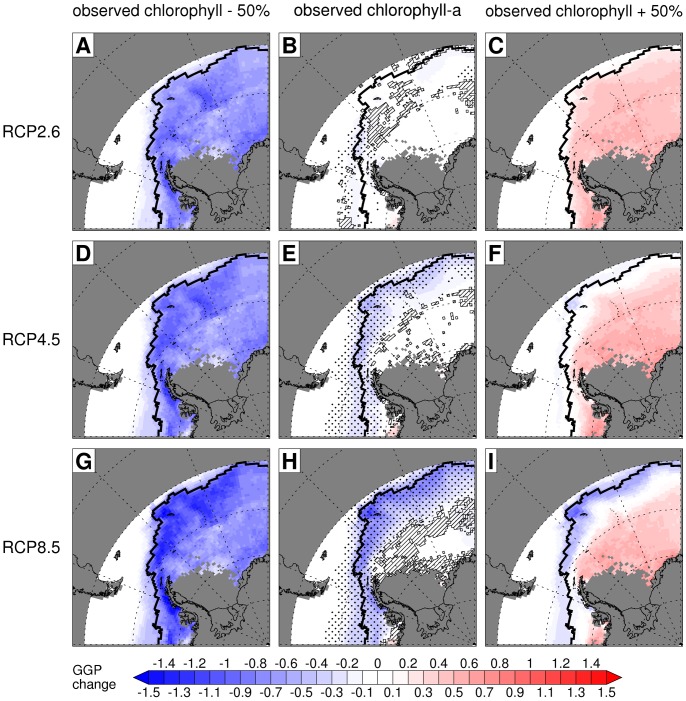
Spatial pattern of projected change in Antarctic krill habitat by the late 21^st^ Century. Each panel shows the projected GGP change (GGP for the period 2070–2099 minus estimated current GGP, as shown in Fig. 2B) calculated across multiple climate models for an Antarctic krill starting length of 40 mm. The GGP values were calculated using bias-corrected SSTs from RCP2.6 (A, B & C), RCP4.5 (D, E & F) or RCP8.5 (G, H & I) and observed chlorophyll-a concentrations reduced by 50% (A, D & G), observed chlorophyll-a concentrations (B, E & H), or observed chlorophyll-a concentrations increased by 50% (C, F & I). Additional symbols (B, E & H) indicate the level of agreement between climate models. Cells where fewer than 50% of the models project significant change (t-test, P≤0.05) from the current period have no additional symbol. Cells where 50% or more of the models project significant change are highlighted with stippling if 90% or more of models agree on the sign of the change, and are highlighted with hatched lines if fewer than 90% agree. The spatial resolution is 1° longitude by 0.5° latitude and the thick black line indicates the northern extent of the current growth area (Fig. 2B).

Projected GGP declines were concentrated in a band that approximates the location of the Antarctic Circumpolar Current (ACC) (Fig. 4). Most models projected significant warming of the ACC under RCP4.5 and RCP8.5. The RCP2.6 results identified an area of warming in the west Scotia Sea, but otherwise there was little agreement between the models about projected changes in GGP under RCP2.6. In the cells where ≥90% of model projections agreed on the sign of change in GGP, the projected GGP declines (with unchanged chlorophyll-a concentrations) were 16%, 25% and 37% for RCP2.6, RCP4.5 and RCP8.5 respectively. The corresponding reductions in growth area were 13%, 23% and 33%. The projections included moderate increases in habitat quality on the continental coast in the far west of the study area, but <90% of model projections agreed on the sign of change in GGP for much of this area.

**Figure 5:**
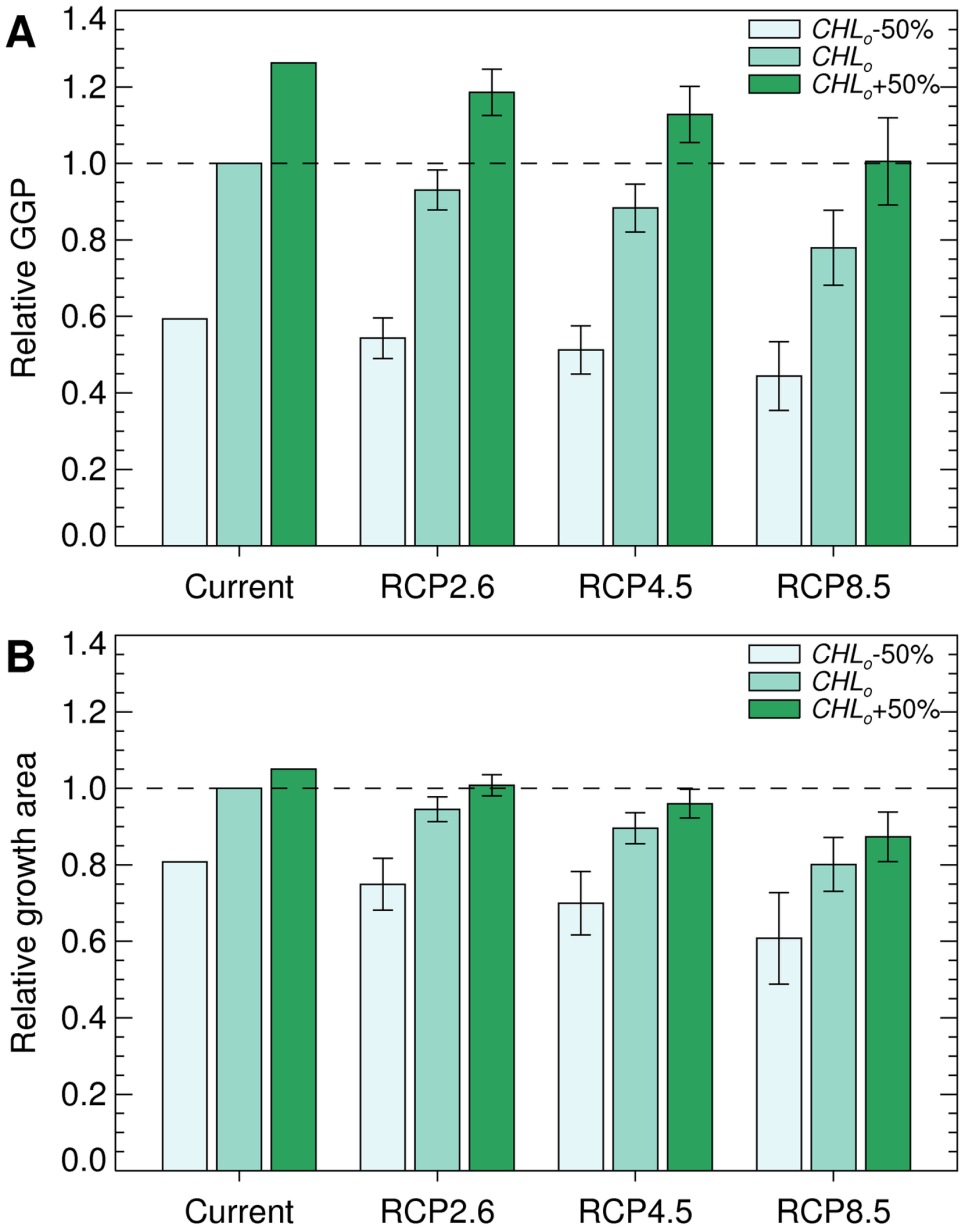
Projected change in Antarctic krill habitat in the study area by the late 21^st^ Century. Relative GGP (GGP for the period 2070–2099 divided by estimated current GGP) (A) and relative growth area (growth area for the period 2070–2099 divided by estimated current growth area) (B), calculated for the study area (Fig. 1B). Results were calculated across multiple models using bias-corrected SSTs from RCP2.6, RCP4.5 or RCP8.5, and observed chlorophyll-a concentrations reduced by 50% (*CHL_o_*-50%), observed chlorophyll-a concentrations (*CHL_o_*), or observed chlorophyll-a concentrations increased by 50% (*CHL_o_*+50%). The assumed Antarctic krill starting length was 40 mm and the error bars show the between-model standard deviation.

The projected GGP declines (with unchanged chlorophyll-a concentrations) across all cells were 7%, 12% and 22% for RCP2.6, RCP4.5 and RCP8.5 respectively (Fig. 5). The corresponding reductions in Antarctic krill growth area were 5%, 10% and 20% for RCP2.6, RCP4.5 and RCP8.5 respectively. Inevitably a 50% reduction in chlorophyll-a concentration led to greater reductions in both GGP and growth area. When chlorophyll-a concentration was increased by 50%, the projected warming under RCPs 4.5 and 8.5 still led to significant reductions in growth area. Nonetheless, the effect of a 50% increase in chlorophyll-a concentration moderated the overall effects of warming on relative GGP.

The spatially-resolved projected GGPs for each combination of RCP and chlorophyll-a concentration had similar maxima which increased slightly (from 4.9 to 5.2) with chlorophyll-a concentration (Fig. S4). The mode of estimated current GGP was 2.1, and the modes of projected GGP with unchanged chlorophyll-a concentrations were similar (1.8 to 2.0) for all RCPs. The mode increased (from about 1.3 to about 2.5) with increasing chlorophyll-a concentration and the area with near modal values declined with increasing SST (i.e. from RCP2.6 to RCP8.5).

**Figure 6:**
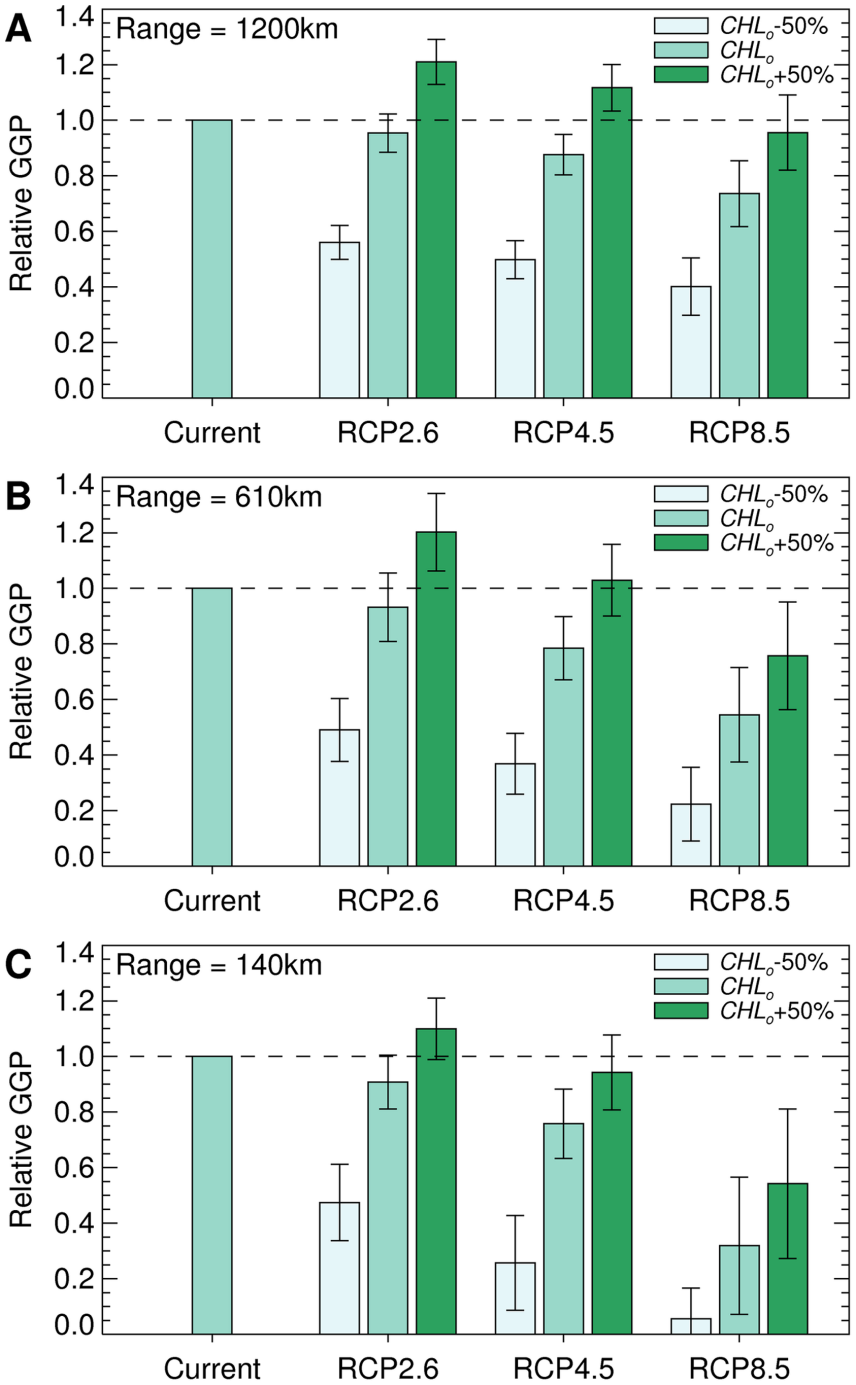
Projected change in Antarctic krill habitat accessible to predators foraging from South Georgia. Relative GGP (GGP for the period 2070–2099 divided by estimated current GGP), calculated within the area accessible to predators with foraging ranges of 1200 km (A), 610 km (B) and 140 km (C) from South Georgia. Results were calculated across multiple models using projected SSTs from RCP2.6, RCP4.5 or RCP8.5, and observed chlorophyll-a concentrations reduced by 50% (*CHL_o_*-50%), observed chlorophyll-a concentrations (*CHL_o_*), or observed chlorophyll-a concentrations increased by 50% (*CHL_o_*+50%). The assumed Antarctic krill starting length was 40 mm and the error bars show the between-model standard deviation.

South Georgia is located in the band of projected GGP declines. Consequently there were pronounced negative effects within the foraging ranges of predators breeding on this island. These negative effects were greatest for those predators with the most restricted foraging ranges (Fig. 6), where the projected GGP declines (with unchanged chlorophyll-a concentrations) were 9%, 24% and 68% and the corresponding reductions in growth area were 5%, 6% and 55% for RCP2.6, RCP4.5 and RCP8.5. The negative effects projected for RCP8.5 were apparent even with the combination of the long foraging range of grey headed albatrosses and an increase in chlorophyll-a concentration.

Analysis of variance (ANOVA) indicated that RCP, chlorophyll-a concentration and climate model each significantly influenced the results shown in Figs. 5 and 6 (e.g. for Fig. 5A three-way ANOVA suggests that each of these factors had a significant influence on the response variable, GGP: F = 187, 3182, 33, P<<0.001). There were also significant interactions between model and chlorophyll-a concentration (F = 4, P<0.001 for Fig. 5A), and model and RCP (F = 2, P = 0.026 for Fig. 5A), which confirms the high degree of between-model variability shown in Figs. 3 and 4. Tukey multiple comparisons tests identified consistent differences (P<0.05) between the results (GGP and growth area) for RCP8.5 and those for RCP2.6 or RCP4.5 when chlorophyll-a concentrations were set at observed levels or observed levels plus 50% (see Table S1, for full results).

## Discussion

The projected effects of plausible SST warming on Antarctic krill growth habitat are mainly negative. Under all RCPs that we considered, the projections imply a decrease in habitat quality over the 21^st^ century, particularly in the ACC. Our analysis suggests that these effects could be mitigated to some extent if warming leads to an overall increase in chlorophyll production. Habitat quality could improve in some marine areas close to the Antarctic continent even under the most extreme warming scenario. However this is unlikely to mitigate the negative impacts within the foraging ranges of birds and seals breeding at South Georgia.

Recent SST warming rates at South Georgia and the western Antarctic Peninsula [6], [7] are in the upper range of projected regional warming rates for the 21^st^ century. However, some parts of the Southern Ocean have cooled over recent decades and experienced associated increases in sea ice [46]. Previous modelling studies suggest that recent warming might have already degraded Antarctic krill habitat in some areas [13], [33]. Analysis of the growth model used here with additional parameters from [34] concluded that, for the period 1970–2004, increasing temperatures probably reduced the lifetime biomass production of Antarctic krill at South Georgia but increased it at the Antarctic Peninsula [33]. Our results suggest that, in the future, increasing temperature could reduce growth in both of these areas. Reduced growth could also affect egg production as smaller females produce fewer eggs [47].

Climate affects species through their habitats. Understanding these habitat effects is a prerequisite for understanding effects on biological variables such as abundance and biomass production. There are many routes through which changing habitats can influence these variables. For example, successful completion of the Antarctic krill life cycle apparently requires spawning in water with specific depth and temperature characteristics [11], [48] and larval development under sea ice [9]. The summer months that we model encompass the main growth period of adult Antarctic krill [11], but winter processes also affect habitat suitability. Other environmental variables, such as pH, might also be critical for the sensitive larval stages [10]. Furthermore, the distribution of Antarctic krill seems to be affected by ocean currents which may transport individuals thousands of kilometres in a lifetime [49]. Thus, high quality growth habitat will only result in high biomass production if sufficient Antarctic krill arrive in the area as a result of transport or local spawning.

These multiple environmental influences on Antarctic krill abundance and biomass production have several implications. Firstly, more detailed mechanistic life-cycle and population models are needed to better assess the potential effects of climate change [9], [38]. For example, a fuller assessment of temperature effects might consider how the relationship between SST and the temperatures that Antarctic krill experience in the water column changes over time and space. Secondly, the environmental effects are likely to be more complex than a simple poleward shift in distribution. Some of the oceanographic characteristics on which Antarctic krill rely, such as the deep waters of the ACC, will not move south as the ocean warms. Coastal embayments and high latitude shelves may be reasonable refugia for growth, but they are unlikely to provide appropriate habitats for spawning [48] or connecting subpopulations [50].

Large scale analyses of ecological responses to climate change generally stress the effects of warming (e.g. [1], [2]). Polar studies also tend to emphasise warming because polar organisms are sensitive to temperature [51], [52], which is rising rapidly in some polar regions [6], [7], [53]. Nonetheless, food availability is also an important habitat characteristic which, at the physiological level, can sometimes compensate for the negative effects of temperature [29]. This is illustrated by the high Antarctic krill abundances and growth rates found at South Georgia. This is near the northern limit of the species’ range and has relatively high and physiologically stressful temperatures, but it also has very high food concentrations (Fig. 1, [12]). Temperature-food interactions are therefore likely to influence the ecological effects of climate change [29]. Using models, such as ours, that explicitly include the effects of food availability is a useful step towards fuller consideration of the multiple interacting effects of climate change [38].

Previous studies have reported a 10% decline in chlorophyll-a concentration in the Southern Ocean over the 1980s and 1990s [40] and substantial localised increases and decreases in chlorophyll-a concentration at the Antarctic Peninsula in the last 30 years [54]. Such changes are associated with changes in the composition of the phytoplankton community. The main effects at the Antarctic Peninsula were an overall decline in chlorophyll-a concentration and a decrease in the abundance of diatoms relative to other phytoplankton [54]. Such changes are consistent with the expected widespread consequences of marine warming [54], [55]. A reduction in diatoms in the diet of Antarctic krill is likely to reduce both growth and reproduction [56], [57]. Therefore the most likely effects of plausible changes in chlorophyll-a concentration are in the range between our reduced and unchanged chlorophyll scenarios.

Models such as those which produced the CMIP5 results are being increasingly used to investigate climate impacts on marine species [38]. These models have many uncertainties, including regional biases and differences between models. Our results confirm a regional bias in Southern Ocean SST [58]. The representation of some Southern Ocean features, such as the ACC, has improved in the CMIP5 results compared to the previous generation of model results [59]. Nonetheless, the available models generally perform badly at reproducing sea ice conditions which, in turn, influence SST [58], [60]. The models differ markedly from each other in terms of both the magnitude and spatial distribution of projected SST changes. We have followed recommended practice for controlling and assessing the influence of these model uncertainties on our results [38]. It is clear from Fig. 4 that most models are in agreement that the ACC will experience significant warming.

Any degradation of Antarctic krill growth habitat in the ACC is likely to have consequences for predators at South Georgia. Analysis of foodweb models suggests that predators able to take advantage of copepod production might be relatively unaffected by a severe reduction in Antarctic krill availability, but that the majority of air-breathing predator populations at South Georgia would probably experience significant declines [15].

The Antarctic krill fishery took 68% of its total catch between 1980 and 2011 from the area of projected severe habitat degradation [18]. Future climate change could therefore have a significant negative effect on Southern Ocean ecosystem services as well as biodiversity. A recommendation that the Commission for the Conservation of Antarctic Marine Living Resources, which is responsible for managing the Antarctic krill fishery, should increase consideration of climate change impacts in its management decisions was made in 1992 [61] but it was not until 2009 that the Commission resolved to do so (www.ccamlr.org/en/resolution-30/xxviii-2009). We suggest that there is a need for more rapid progress in developing methods for evaluating climate change impacts in parallel with improved regional climate projections, and for adaptation to and management of the risks to the Southern Ocean ecosystem that climate change implies.

## Supporting Information

Figure S1 **Projected change in Antarctic krill habitat based on a starting length of 30 mm.** Each panel shows the projected GGP change (GGP for the period 2070–2099 minus estimated current GGP) calculated across multiple climate models. The assumed Antarctic krill starting length was 30 mm. The GGP values were calculated using bias-corrected SSTs from RCP2.6 (A, B & C), RCP4.5 (D, E & F) or RCP8.5 (G, H & I) and observed chlorophyll-a concentrations reduced by 50% (A, D & G), observed chlorophyll-a concentrations (B, E & H), or observed chlorophyll-a concentrations increased by 50% (C, F & I). The spatial resolution is 1° longitude by 0.5° latitude and the thick black line indicates the boundaries of the growth area for that panel.
(TIFF)

Figure S2 **Projected change in Antarctic krill habitat based on a starting length of 40 mm.** Each panel shows the projected GGP change calculated across multiple climate models. The assumed Antarctic krill starting length was 40 mm. Other details as Fig. S1.
(TIFF)

Figure S3 **Projected change in Antarctic krill habitat based on a starting length of 50 mm.** Each panel shows the projected GGP change calculated across multiple climate models. The assumed Antarctic krill starting length was 50 mm. Other details as Fig. S1.
(TIFF)

Figure S4 **Distribution of GGP values in the results presented in** **Figs. 4** **and** **5** **.** Each panel shows the distribution of projected GGP values for the period 2070–2099 as the percent coverage of the modelled area (coloured lines). The projected GGP values were calculated using bias-corrected SSTs from RCP2.6 (A, B & C), RCP4.5 (D, E & F) or RCP8.5 (G, H & I). The panels also show the distribution of estimated GGP values calculated using observed SSTs (for the period 2002–2011) (grey bars). Both sets of GGP values in each panel were calculated using the same chlorophyll-a concentrations: observed chlorophyll-a concentrations reduced by 50% (A, D & G), observed chlorophyll-a concentrations (B, E & H), or observed chlorophyll-a concentrations increased by 50% (C, F & I).
(TIFF)

Table S1 **Statistical comparison of scenario-specific results presented in** **Figs. 5** **and** **6** **.** The table shows the probability (from Tukey multiple comparisons tests) that the projected GGP or growth area for each combination of chlorophyll-a concentration and RCP is significantly different from comparable results for other RCPs. We compared each result shown in Figs. 5 and 6 with the other results in the same figure panel. Comparisons which were significantly different (P<0.05) are highlighted in bold text.
(DOC)
