# Supplementary figures and images for: Potential Climate Change Effects on the Habitat of Antarctic Krill in the Weddell Quadrant of the Southern Ocean
Source: PLoS One. 2013 Aug 21;8(8):e72246. doi: 10.1371/journal.pone.0072246 (PMC3749108; doi:10.1371/journal.pone.0072246)

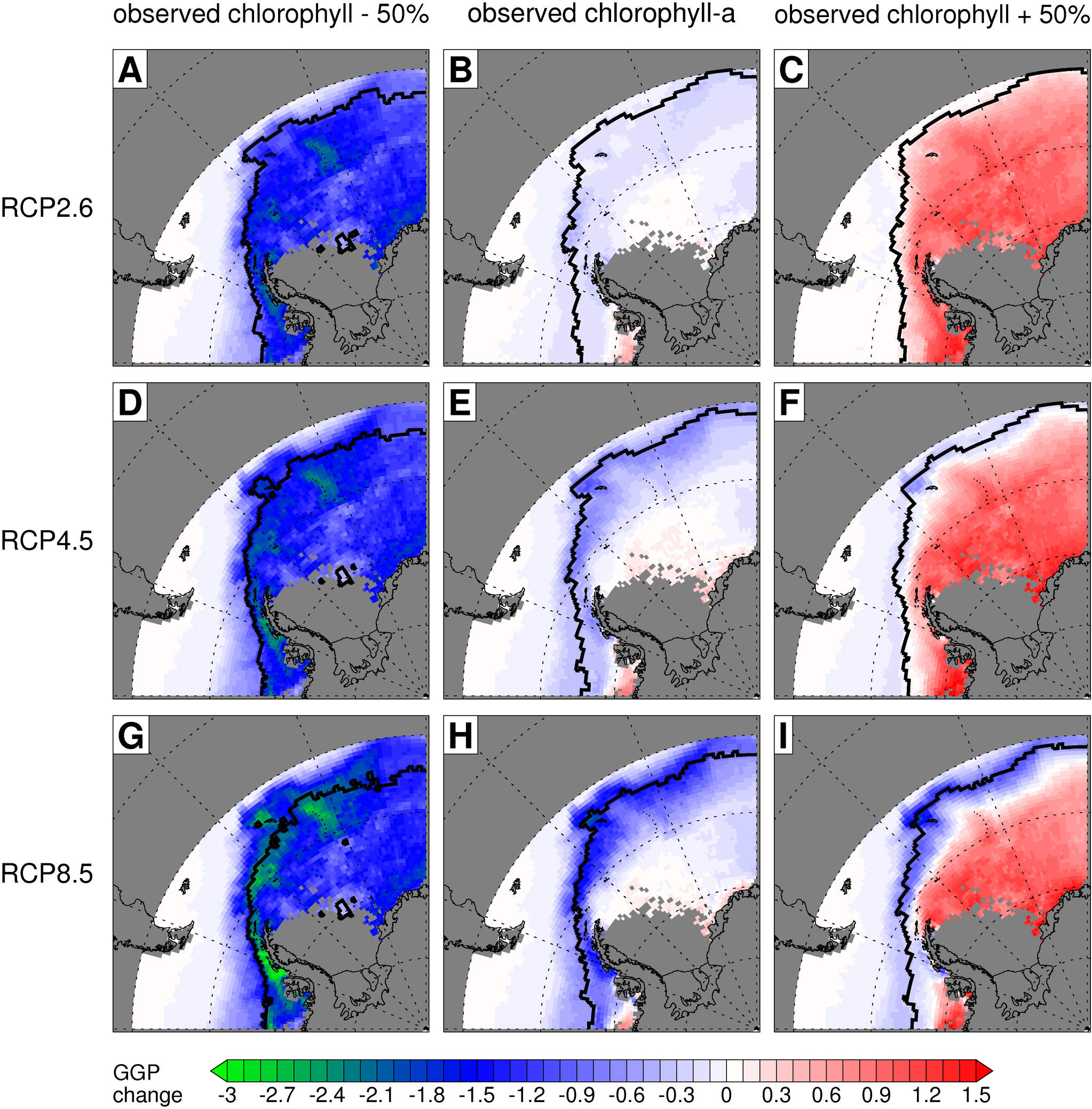

Supplement: Figure S1 — Projected change in Antarctic krill habitat based on a starting length of 30 mm. Each panel shows the projected GGP change (GGP for the period 2070–2099 minus estimated current GGP) calculated across multiple climate models. The assumed Antarctic krill starting length was 30 mm. The GGP values were calculated using bias-corrected SSTs from RCP2.6 (A, B & C), RCP4.5 (D, E & F) or RCP8.5 (G, H & I) and observed chlorophyll-a concentrations reduced by 50% (A, D & G), observed chlorophyll-a concentrations (B, E & H), or observed chlorophyll-a concentrations increased by 50% (C, F & I). The spatial resolution is 1° longitude by 0.5° latitude and the thick black line indicates the boundaries of the growth area for that panel. (TIFF) [file pone.0072246.s001.tiff]

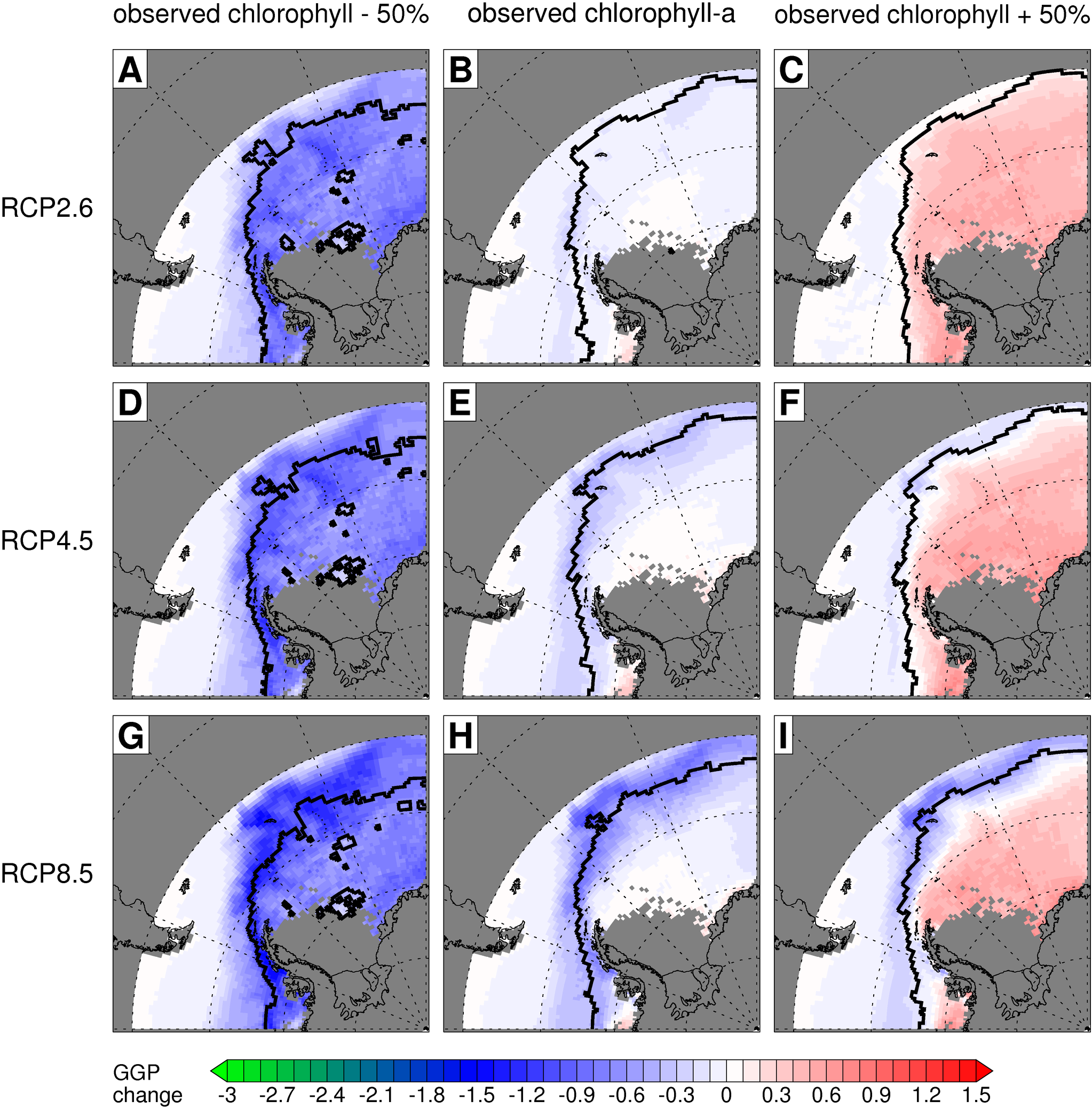

Supplement: Figure S2 — Projected change in Antarctic krill habitat based on a starting length of 40 mm. Each panel shows the projected GGP change calculated across multiple climate models. The assumed Antarctic krill starting length was 40 mm. Other details as Fig. S1. (TIFF) [file pone.0072246.s002.tiff]

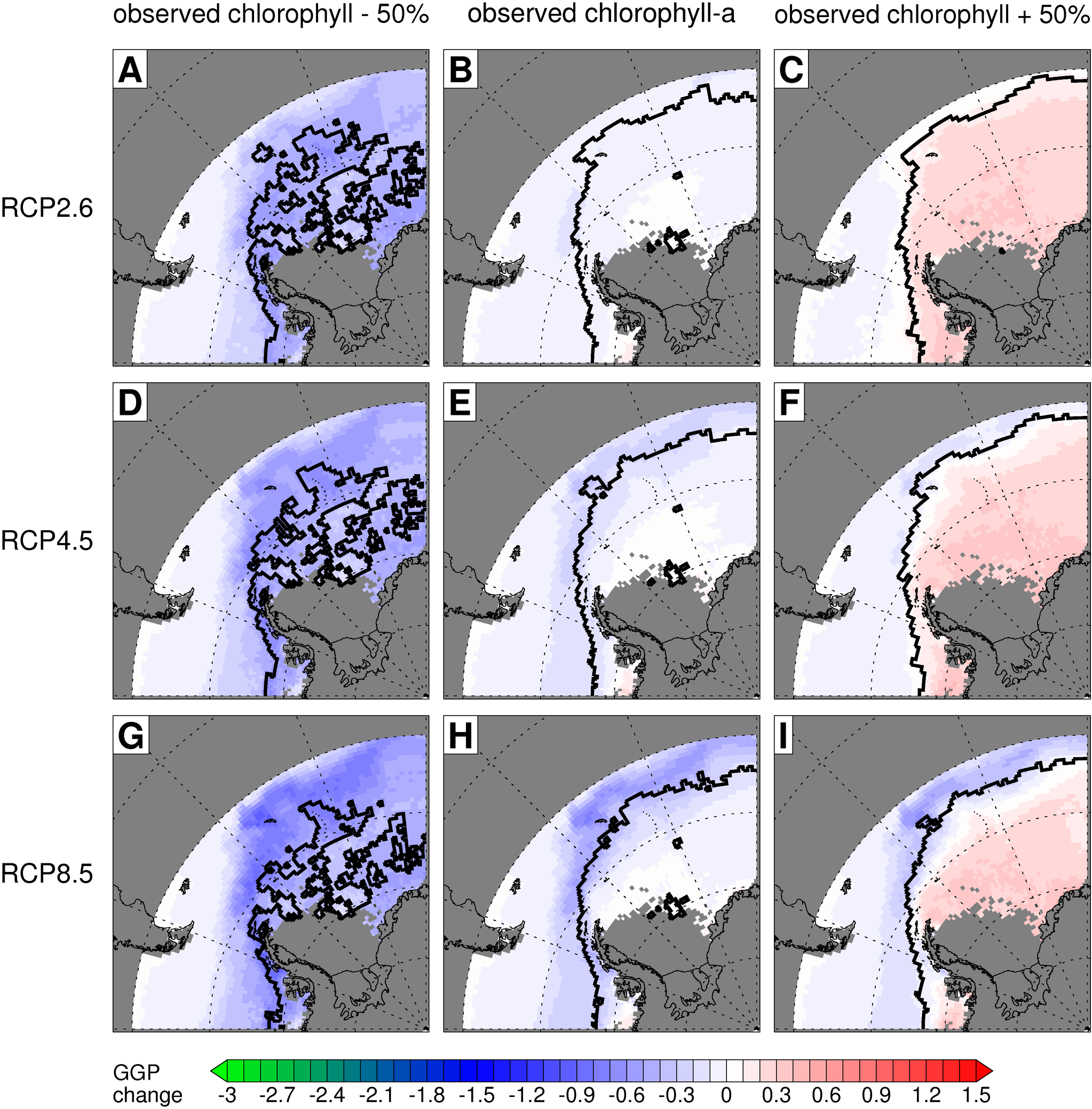

Supplement: Figure S3 — Projected change in Antarctic krill habitat based on a starting length of 50 mm. Each panel shows the projected GGP change calculated across multiple climate models. The assumed Antarctic krill starting length was 50 mm. Other details as Fig. S1. (TIFF) [file pone.0072246.s003.tiff]

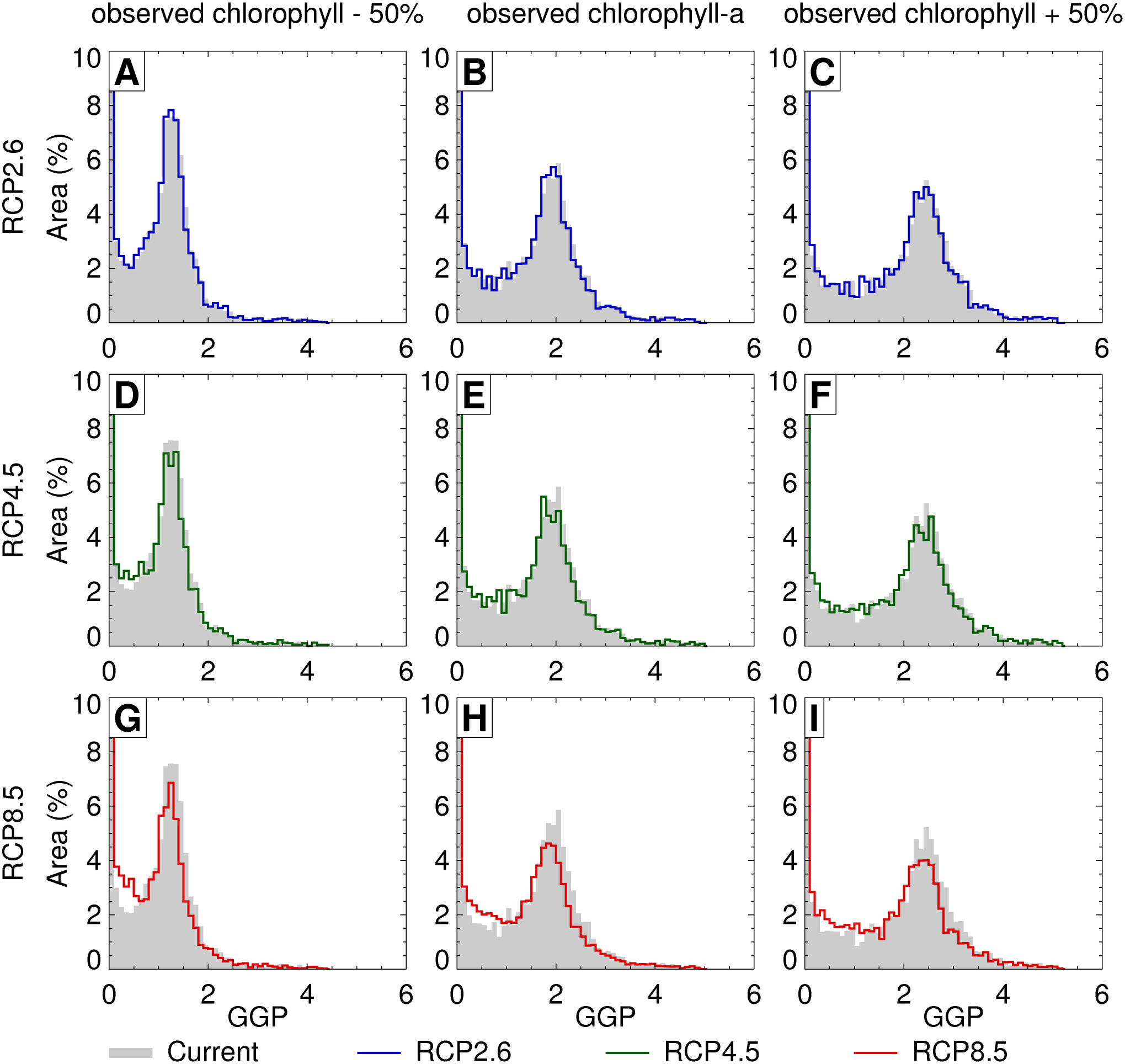

Supplement: Figure S4 — Distribution of GGP values in the results presented in Figs. 4 and 5 . Each panel shows the distribution of projected GGP values for the period 2070–2099 as the percent coverage of the modelled area (coloured lines). The projected GGP values were calculated using bias-corrected SSTs from RCP2.6 (A, B & C), RCP4.5 (D, E & F) or RCP8.5 (G, H & I). The panels also show the distribution of estimated GGP values calculated using observed SSTs (for the period 2002–2011) (grey bars). Both sets of GGP values in each panel were calculated using the same chlorophyll-a concentrations: observed chlorophyll-a concentrations reduced by 50% (A, D & G), observed chlorophyll-a concentrations (B, E & H), or observed chlorophyll-a concentrations increased by 50% (C, F & I). (TIFF) [file pone.0072246.s004.tiff]
