# Supplementary material for: Potential Climate Change Effects on the Habitat of Antarctic Krill in the Weddell Quadrant of the Southern Ocean
Source: PLoS One. 2013 Aug 21;8(8):e72246. doi: 10.1371/journal.pone.0072246 (PMC3749108; doi:10.1371/journal.pone.0072246)
Supplement: Table S1 — Statistical comparison of scenario-specific results presented in Figs. 5 and 6 . The table shows the probability (from Tukey multiple comparisons tests) that the projected GGP or growth area for each combination of chlorophyll-a concentration and RCP is significantly different from comparable results for other RCPs. We compared each result shown in Figs. 5 and 6 with the other results in the same figure panel. Comparisons which were significantly different (P<0.05) are highlighted in bold text. (DOC) [file pone.0072246.s005.doc]

| Comparison | | | Fig. 5 | | Fig. 6 | | |
| --- | --- | --- | --- | --- | --- | --- | --- |
|  |  |  |  |  | (A) 1200 km | (B) 610 km | (C) 140 km |
| Chlorophyll-a concentration | 1^st^ RCP | 2^nd^ RCP | Growth area | GGP | GGP | GGP | GGP |
| *CHL_o_*-50% | RCP8.5 | RCP4.5 | **0.007** | **0.021** | 0.094 | 0.099 | **0.032** |
| *CHL_o_*-50% | RCP8.5 | RCP2.6 | **0.000** | 0.282 | **0.000** | **0.000** | **0.000** |
| *CHL_o_*-50% | RCP4.5 | RCP2.6 | 0.567 | 0.979 | 0.681 | 0.327 | **0.020** |
| *CHL_o_* | RCP8.5 | RCP4.5 | **0.005** | **0.010** | **0.001** | **0.000** | **0.000** |
| *CHL_o_* | RCP8.5 | RCP2.6 | **0.000** | **0.000** | **0.000** | **0.000** | **0.000** |
| *CHL_o_* | RCP4.5 | RCP2.6 | 0.559 | 0.787 | 0.374 | 0.115 | 0.300 |
| *CHL_o_*+50% | RCP8.5 | RCP4.5 | **0.014** | **0.001** | **0.000** | **0.000** | **0.000** |
| *CHL_o_*+50% | RCP8.5 | RCP2.6 | **0.000** | **0.000** | **0.000** | **0.000** | **0.000** |
| *CHL_o_*+50% | RCP4.5 | RCP2.6 | 0.602 | 0.548 | 0.157 | **0.031** | 0.241 |
